# Solvent Composition is Critical for Carbodiimide Cross-Linking of Hyaluronic Acid as an Ophthalmic Biomaterial

**DOI:** 10.3390/ma5101986

**Published:** 2012-10-23

**Authors:** Jui-Yang Lai

**Affiliations:** 1 Institute of Biochemical and Biomedical Engineering, Chang Gung University, Taoyuan 33302, Taiwan; E-Mail: jylai@mail.cgu.edu.tw; Tel.: +886-3-211-8800, ext. 3598; Fax: +886-3-211-8668; 2 Biomedical Engineering Research Center, Chang Gung University, Taoyuan 33302, Taiwan; 3 Molecular Medicine Research Center, Chang Gung University, Taoyuan 33302, Taiwan

**Keywords:** hyaluronic acid, carbodiimide, cross-linking, solvent composition, ophthalmic biomaterial

## Abstract

Hyaluronic acid (HA) is one of the most important ophthalmic biomaterials, while also being used for tissue engineering and drug delivery. Although chemical cross-linking is an effective way to improve the material performance, it may as a consequence be detrimental to the living cells/tissues. Given that the cross-linking efficiency is mediated by the solvent composition during the chemical modification, this study aims to explore the stability and biocompatibility of carbodiimide cross-linked HA in relation to material processing conditions by varying the acetone/water volume ratio (from 70:30 to 95:5) at a constant 1-ethyl-3-(3-dimethyl aminopropyl) carbodiimide (EDC) concentration of 100 mM. Our results indicated that after the EDC treatment in the presence of an acetone/water mixture (85:15, v/v), the HA hydrogel membranes have the lowest equilibrium water content, the highest stress at break and the greatest resistance to hyaluronidase digestion. Live/Dead assays and pro-inflammatory cytokine expression analyses showed that the cross-linked HA hydrogel membranes, irrespective of the solvent composition, are compatible with human RPE cell lines without causing toxicity and inflammation. However, it should be noted that the test samples prepared by the cross-linking in the presence of acetone/water mixtures containing 70, 75, and 95 vol % of acetone slightly inhibit the metabolic activity of viable ARPE-19 cultures, probably due to the alteration in the ionic interaction between the medium nutrients and polysaccharide biomaterials. In summary, the water content, mechanical strength and RPE cell proliferative capacity strongly depends on the solvent composition for carbodiimide cross-linking of HA materials.

## 1. Introduction

Due to the unique anatomy and physiology of the eye, the development of drug delivery systems for posterior segment ocular diseases remains full of challenges. Intravitreal injection of drugs has received much attention given that this route of administration achieves high drug levels at the site of action (*i.e.*, vitreous and retina) and avoids adverse effects caused by systemic administration [1]. Over the past few years, advances in biomaterials science have made tremendous improvements in this area. A study from Chang-Lin *et al.* demonstrated that the poly(lactic acid-*co*-glycolic acid) implants containing dexamethasone, approved by the United States Food and Drug Administration, could be given as a sustained-release formulation for the intravitreal treatment of macular edema after branch or central retinal vein occlusion, and for the treatment of noninfectious uveitis affecting the posterior segment of the eye [2]. Wells *et al.* also prepared photoresponsive polyethylene glycol-anthracene grafted hyaluronan that was compatible with retinal pigment epithelial cell lines and able to deliver a variety of model compounds (*i.e.*, anti-inflammatory steroids and vascular endothelial growth factor-blocker drugs) over the long term (months) [3]. More recently, Bochot *et al.* reported the potential of liposomes for improving the treatment of cytomegalovirus-induced retinitis in humans by reducing the toxicity and increasing the residence time of several poorly-stable drugs such as peptides and nucleic acids in the eye [4].

Hyaluronic acid (HA) is a linear anionic polysaccharide comprised of repeating disaccharide units of d-glucuronic acid and *N*-acetyl-d-glucosamine. As one of the main components of the extracellular matrix, HA has been used as a coating material for cultivation of corneal keratocyte spheroids in our laboratory [5]. Due to its high capacity for lubrication, water sorption and water retention, HA-based hydrogel is particularly attractive for skin and cartilage tissue engineering applications [6,7]. The unique characteristics of HA also make it a promising candidate in clinical ophthalmology: it can be employed as an artificial tear ingredient for the treatment of dry eyes, and a viscoelastic agent for cataract surgery and deep lamellar keratoplasty [8,9,10]. In 2006, Suri *et al.* evaluated the feasibility of using biopolymers composed of gellan and HA as *in situ* gels for short term vitreous substitution and concluded that the biomaterials may have similar biophysical properties to vitreous and may become promising alternatives to silicone oil [11]. In this respect, investigators are encouraged to consider the further development of HA-based biopolymers as drug delivery carriers for the treatment of posterior segment ocular diseases.

It has been documented that the HA molecule has a short residence time in tissue (usually a few days) [12]. Chemical modification techniques have been applied to overcome the rapid degradation of HA materials. In addition to derivatization method [13], cross-linking approach is another powerful strategy to tune the physicochemical properties of HA [14,15]. Various chemical cross-linkers such as glutaraldehyde [16], epoxy compound [17], carbodiimide [18,19] and divinyl sulfone [20] have been described for the creation of intermolecular covalent bonds, thereby contributing to the stability of HA chains. Among these cross-linking agents, 1-ethyl-3-(3-dimethyl aminopropyl) carbodiimide hydrochloride (EDC) is preferable because it can induce cross-linking of biomaterials without taking part in the linkages but simply change to water-soluble urea derivatives that have very low cytotoxicity [21]. Therefore, our previous studies validate EDC as a potential biopolymer cross-linker for the fabrication of various chemically modified carriers/scaffolds for ocular tissue engineering [22,23,24].

During the process of cross-linking, solvent concentration plays an important role on the extent of cross-linking of the resulting biomaterials. Tomihata *et al.* have investigated the effect of ethanol concentration in the reaction medium (*i.e.*, ethanol/water mixtures) on EDC cross-linking of gelatin films in the heterogeneous state and concluded that minimum water content is attained at an ethanol concentration of around 80 vol % [25]. A suitable organic solvent, such as acetone, can also prevent the deactivation of water-soluble carbodiimide since this type of cross-linker easily loses its activity in an aqueous solution. Choi *et al.* have prepared insoluble gelatin-alginate sponges by cross-linking of the samples in an acetone/water mixture (9:1, v/v) containing 20–100 mg of EDC [26]. The same solvent system has been adopted by us in the cross-linking of HA hydrogel discs [18]. Given that the structural stability, degradation rate and biocompatibility of chemically modified biomaterials strongly depends on their cross-linking degree [23,27], the present work aims to investigate the influence of solvent composition-mediated cross-linking on the water content, mechanical strength and *in vitro* degradability of EDC cross-linked HA hydrogels. The *in vitro* biocompatibility of HA membranes treated with cross-linker (100 mM EDC) in the presence of binary acetone/water mixtures of varying acetone concentrations (70–95 vol %) was analyzed using human retinal pigment epithelial (RPE) cell line cultures. The cell viability and pro-inflammatory cytokine expression were studied to give insight into the role of solvent concentration on cellular responses to biomaterials. In addition, after three days of culture with the test samples, RPE cell growth was examined to clarify the relationship between the extent of cross-linking of the HA and cellular proliferative capacity.

## 2. Experimental Section

### 2.1. Materials

Hyaluronic acid sodium salt was obtained from Kewpie (Tokyo, Japan) as a dry powder. It was made by fermentation method and was highly purified. According to information from the supplier, the HA samples used in this study had a weight-average molecular weight of around 1100 kDa. 1-ethyl-3-(3-dimethyl aminopropyl) carbodiimide hydrochloride (EDC) and hyaluronidase type V from sheep testes (1770 units/mg) were purchased from Sigma-Aldrich (St. Louis, MO, USA). Deionized water used was purified with a Milli-Q system (Millipore, Bedford, MA, USA). Balanced salt solution (BSS, pH 7.4) was obtained from Alcon Laboratories (Fort Worth, TX, USA). Phosphate-buffered saline (PBS, pH 7.4) was obtained from Biochrom AG (Berlin, Germany). Dulbecco’s modified Eagle’s medium/Ham’s F12 nutrient mixture (DMEM/F12) was purchased from Gibco-BRL (Grand Island, NY, USA). Fetal bovine serum (FBS) and the antibiotic/antimycotic (A/A) solution (10,000 U/mL penicillin, 10 mg/mL streptomycin and 25 μg/mL amphotericin B) were obtained from Biological Industries (Kibbutz Beit Haemek, Israel). All the other chemicals were of reagent grade and used as received without further purification.

### 2.2. Carbodiimide Cross-Linking of HA Membranes

The HA hydrogels were prepared by solution casting methods as we have described elsewhere [18,22]. Briefly, 0.5 wt % aqueous solution of HA was poured into a polystyrene planar mold (5 × 5 cm^2^, 1.5 cm depth), and air-dried for three days at 25 °C to obtain hydrogel sheets (approximately 100 μm in thickness).

The HA samples were subsequently cross-linked by immersing the hydrogels in binary solvent mixtures containing cross-linking agent (100 mM EDC). In acetone/water mixtures, the acetone concentrations varied from 70 to 95 vol %. The cross-linking reaction was allowed to proceed at 25 °C for two days. The resulting samples were thoroughly washed with deionized water to remove excess EDC and urea by-product, and were dried *in vacuo* for 24 h. In this study, the HA material cross-linked in an acetone/water mixture (95:5, v/v) was designated as A95W05.

### 2.3. Extent of Cross-Linking

The water content measurements were used to estimate the extent of cross-linking of each HA membrane. The test samples were first dried to constant weight (*W*_i_) *in vacuo* and were immersed in deionized water at 25 °C with reciprocal shaking (50 rpm) in a thermostatically controlled water bath. After 6 h, the swollen hydrogel membranes were blotted with tissue paper to remove excess water on the surface, and immediately weighed (*W*_s_). The equilibrium water content (%) of the test sample was defined by ((*W*_s_ − *W*_i_)/*W*_s_) × 100 as described previously [28,29]. Results were averaged on six independent runs.

### 2.4. Mechanical Strength

In the tensile tests, the cross-linked HA membranes were placed in an environment with humidity of 75% for 24 h [27,30]. Then, the dumbbell-shaped samples were prepared by cutting wet membranes under pressure with a suitable mold. The gauge length of the specimens was 10 mm and the width was 5 mm. Sample thicknesses were measured at three different points with a Pocket Leptoskop electronic thickness gauge (Karl Deutsch, Germany) and the average was taken. The stress at break and Young’s modulus values of HA samples were determined using an Instron Mini 44 universal testing machine (Canton, MA, USA). All measurements were performed at 25 °C and a relative humidity of 50% using a crosshead speed of 0.5 mm/min. Results were averaged on eight independent runs.

### 2.5. *In Vitro* Degradability

To measure the extent of degradation, each HA membrane was first dried to constant weight (*W*_i_) *in vacuo*. The test samples were immersed in BSS containing 400 units/mL hyaluronidase and incubated at 37 °C with reciprocal shaking (50 rpm) in a thermostatically controlled water bath. After 36 h, the degraded hydrogels were collected and further dried *in vacuo*. The dry weight of samples after degradation (*W*_d_) was determined and the percentage of weight remaining (%) was calculated as (*W*_d_/*W*_i_) × 100 [31,32]. Results were the average of five independent measurements.

### 2.6. Human RPE Cell Line Cultures

ARPE-19 cells, a spontaneously immortalized human cell line (BCRC No. 60383) with morphological and functional characteristics similar to adult human RPE [33], were purchased from the Bioresource Collection and Research Center (Hsinchu, Taiwan, ROC). The cells were maintained in regular growth medium containing DMEM/F12, 10% FBS, and 1% A/A solution. Cultures were incubated in a humidified atmosphere of 5% CO_2_ at 37 °C. The cells from passage 41 were used for experiments.

For evaluation of cellular responses to cross-linked HA materials, the method was used as described previously [34]. In brief, ARPE-19 cells (7 × 10^4^ cells/well) were seeded in 24-well plates and maintained at 37 °C with 5% CO_2_. Using cell culture inserts (Falcon 3095, Becton Dickinson Labware, Franklin Lakes, NJ, USA), each well of a 24-well plate was divided into two compartments. A membrane sample (1 × 1 cm^2^) was then placed into the inner well of the double-chamber system to examine the cells cultured on the plastic plate. At specific time intervals, the qualitative and quantitative assays were performed following removal of the inserts and HA samples. ARPE-19 cells in regular growth medium without test materials served as control groups.

### 2.7. Cell Viability

ARPE-19 cells were grown to confluence and then were exposed to various EDC cross-linked HA membranes for three days. Cell viability was determined using the Live/Dead Viability/Cytotoxicity Kit from Molecular Probes (Eugene, OR, USA) [35]. This assay uses intracellular esterase activity to identify the living cells; the process cleaves the calcein acetoxymethyl to produce a green fluorescence. Ethidium homodimer-1 can easily pass through the damaged cell membranes of dead cells to bind to the nucleic acids, yielding a red fluorescence. After washing three times with PBS, the cultures were stained with a working solution consisting of 2 μL of ethidium homodimer-1, 1 mL of PBS, and 0.5 μL of calcein acetoxymethyl. Under fluorescence microscopy (Axiovert 200M; Carl Zeiss, Oberkochen, Germany), three different areas each containing approximately 500 cells were counted at 100× magnification. All experiments were performed in triplicate, and the viability of the ARPE-19 cell cultures was expressed as the average ratio of live cells to the total number of cells in these nine different areas.

### 2.8. Pro-Inflammatory Cytokine Expression

ARPE-19 cells were grown to confluence on 24-well plates in regular growth medium. Following a 3-day exposure of cultures to test samples, aliquots of the supernatant were collected to measure the interleukin-6 (IL-6) levels. The release of IL-6 from cultivated cells into the conditioned medium was detected by the Quantikine enzyme-linked immunosorbent assay (ELISA) kit (R&D Systems, Minneapolis, MN, USA) specific for human IL-6. Cytokine bioassays were performed according to the manufacturer’s instructions. Photometric readings at 450 nm were measured using the Spectrophotometer (ThermoLabsystems) [36]. Results were expressed as pg/mL. All experiments were conducted in quadruplicate.

### 2.9. Cell Proliferation

ARPE-19 cells were seeded in 24-well plates containing regular growth medium and incubated overnight to allow cell attachment. Following a 3-day exposure to test samples, the cell growth was estimated using the CellTiter 96 Aqueous Non-Radioactive Cell Proliferation MTS Assay (Promega, Madison, WI, USA), in which MTS tetrazolium compound is bio-reduced by cells to form a water-soluble colored formazan [37]. A total of 100 μL of the combined MTS/PMS (20:1) reagent was added to each well of the 24-well plate, and incubated for 3 h at 37 °C in a CO_2_ incubator. The data of absorbance readings at 490 nm were measured using the Multiskan Spectrum Microplate Spectrophotometer (ThermoLabsystems, Vantaa, Finland). All experiments were performed in five replicates, and the results were expressed as relative MTS activity when compared to control groups.

### 2.10. Statistics

Results were expressed as “mean ± standard deviation (SD)”. Comparative studies of means were performed using one-way analysis of variance (ANOVA). Significance was accepted with *P* < 0.05.

## 3. Results and Discussion

### 3.1. Extent of Cross-Linking

**Figure 1:**
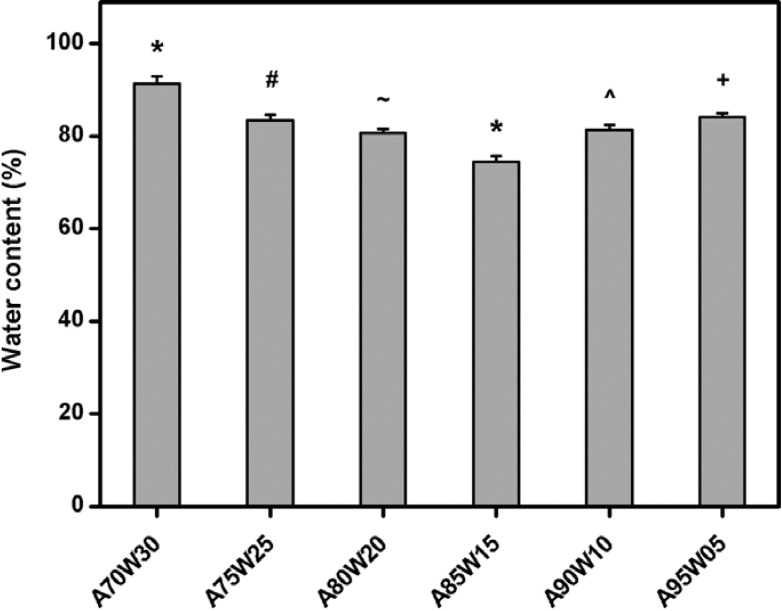
Equilibrium water content of 1-ethyl-3-(3-dimethyl aminopropyl) carbodiimide (EDC) cross-linked hyaluronic acid (HA) membranes as a function of solvent composition. Values are mean ± SD (*n* = 6). *P < 0.05 *vs*. all groups; ^#^*P* < 0.05 *vs*. all groups, except A95W05; ^+^*P* < 0.05 *vs.* all groups, except A75W25; ^~^*P* < 0.05 *vs.* all groups, except A90W10; ^^^*P* < 0.05 *vs.* all groups, except A80W20.

EDC has been reported as a cross-linking agent that is responsible for the intermolecular formation of ester bonds between the hydroxyl and carboxyl groups of HA [18]. The presence of cross-links can reduce the water absorption capability of biopolymer matrices [21]. Hence, in this study, the extent of cross-linking of HA hydrogels was determined by a gravimetric method. Figure 1 shows the water content of various EDC cross-linked HA membranes after incubation in deionized water at 25 °C for 6 h. The samples from A85W15 groups had an equilibrium water content of 74.5% ± 1.2%, which was significantly lower than those of all the other groups (*P* < 0.05). Our results suggest that the formation of the highest amount of cross-links within the HA hydrogels is observed after the EDC treatment in the presence of acetone/water mixture (85:15, v/v). When the acetone concentration in binary solvent mixtures was decreased from 85 to 70 vol %, the water content of the chemically modified HA samples significantly increased (*P* < 0.05). The swelling of HA materials may increase the intermolecular distance, which reduces the collision frequency of polysaccharide molecules with chemical cross-linking agent to create covalent bonds between biopolymer chains. On the other hand, in the range of 85 to 95 vol %, the water content significantly increased with increasing acetone concentration (*P* < 0.05). An incomplete cross-linking in the interior of material samples A90W10 and A95W05 probably occurs due to the poor solubility of water-soluble carbodiimide in solvent of higher fraction of acetone. These findings indicate that the extent of cross-linking of HA hydrogels strongly depends on the solvent composition.

### 3.2. Mechanical Strength

**Figure 2:**
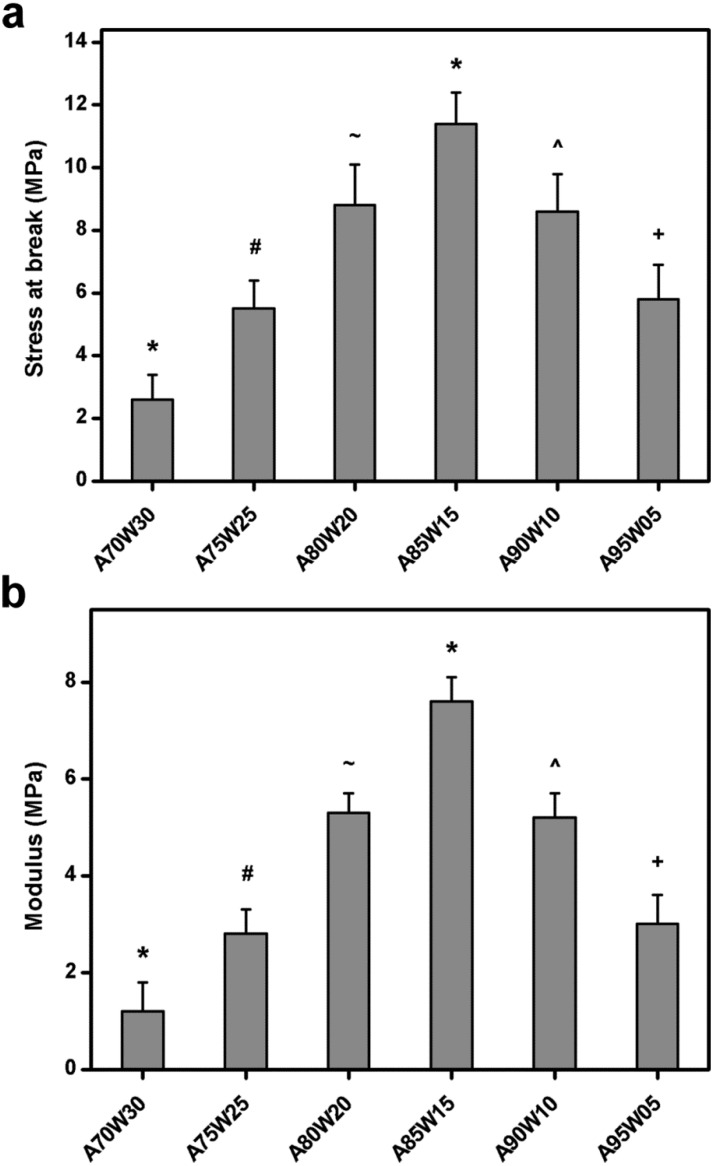
Mechanical properties of EDC cross-linked HA membranes as a function of solvent composition. (**a**) Tensile stress; (**b**) Young’s modulus. Values are mean ± SD (*n* = 8). *P < 0.05 *vs.* all groups; ^#^*P* < 0.05 *vs.* all groups, except A95W05; ^+^*P* < 0.05 *vs.* all groups, except A75W25; ^~^*P* < 0.05 *vs.* all groups, except A90W10; ^^^*P* < 0.05 *vs.* all groups, except A80W20.

The mechanical properties of various EDC cross-linked HA membranes are summarized in Figure 2. The test samples from A70W30, A75W25, A80W20, and A85W15 groups exhibited stress at a break of 2.6 ± 0.8, 5.5 ± 0.9, 8.8 ± 1.3, and 11.4 ± 1.0 MPa, respectively (Figure 2a). The values showed significant differences between these four groups (*P* < 0.05), indicating that the tensile strength increased with increasing acetone concentration in binary solvent mixtures. However, when the acetone concentration was further increased from 85 to 95 vol %, the stress at break of the chemically modified HA samples significantly decreased (*P* < 0.05). A similar trend was found for the effect of solvent composition on Young’s modulus variation (Figure 2b). The present findings support the evaluation of the extent of cross-linking and water content. We have previously shown that the EDC cross-linked HA samples prepared in the presence of acetone/water mixture (80:20, v/v) have a stress at break of 25.7 ± 2.3 MPa [22], which is significantly higher than that obtained in this work. One possible explanation for these observations is that the mechanical behavior of chemically modified HA materials is examined under different conditions. While the dry samples are used for earlier tensile tests, the HA membranes are placed in an environment with humidity of 75% for 24 h prior to testing here. It has been reported that the reduction in tensile stress by plasma sterilization is attributed to the increased water absorption of gelatin-based biopolymer carriers [38]. The presence of absorbed water will also affect the mechanical strength and stability of hydrophilic HA networks.

### 3.3. *In Vitro* Degradability

**Figure 3:**
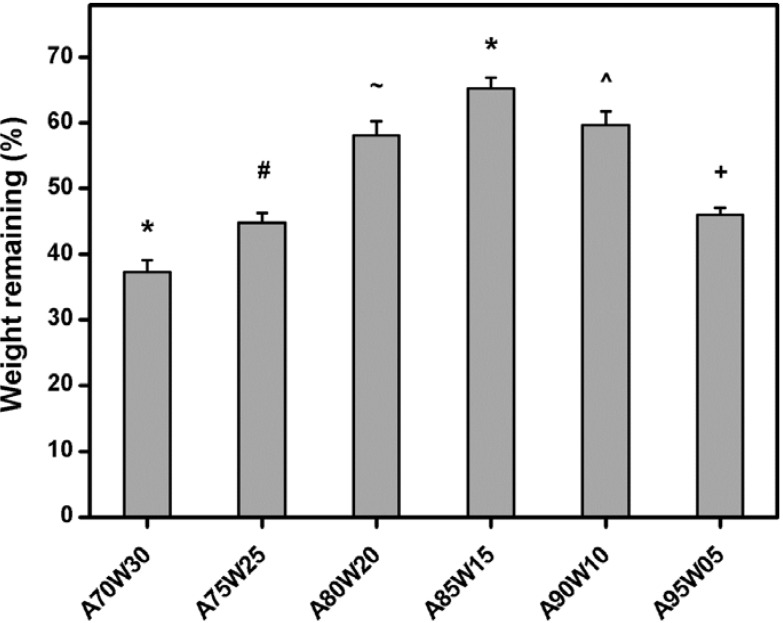
Weight remaining of various EDC cross-linked HA membranes after incubation at 37 °C for 36 h in BSS containing hyaluronidase. Values are mean ± SD (*n* = 5). *P < 0.05 *vs.* all groups; ^#^*P* < 0.05 *vs.* all groups, except A95W05; ^+^*P* < 0.05 *vs.* all groups, except A75W25; ^~^*P* < 0.05 *vs.* all groups, except A90W10; ^^^*P* < 0.05 *vs.* all groups, except A80W20.

HA is a promising biomaterial for the production of hydrogel carriers, since it is degradable under the action of hyaluronidase [39]. Therefore, the *in vitro* degradation behavior of test samples was investigated by incubation at 37 °C in BSS (*i.e.*, physiological medium) containing hyaluronidase. The residual mass percentage of the EDC cross-linked HA membranes as a function of solvent composition is presented in Figure 3. In the A70W30, A75W25, A80W20, A90W10, and A95W05 groups, the weight remaining after 36 h was 37.3% ± 1.8%, 44.8% ± 1.5%, 58.1% ± 2.2%, 59.7% ± 2.1%, and 46.0% ± 1.1%, respectively. These values were significantly lower than those of the A85W15 (65.3% ± 1.7%) groups (*P* < 0.05), indicating that the differences in the *in vitro* degradability of HA membranes modified in the presence of binary acetone/water mixtures may be due to the influence of solvent composition. Segura *et al.* have reported a cross-linking strategy targeting the alcohol groups via a poly(ethylene glycol) diepoxide cross-linker for the generation of degradable HA hydrogels [40]. The time for complete enzymatic degradation of the samples in a 100 units/mL solution of hyaluronidase depends on their equivalents of cross-linker, with the highest cross-linking degree producing the most stable hydrogels. Our results were compatible with these earlier observations, and suggested that the formation of more intermolecular cross-links in the samples with a higher extent of cross-linking decreases the access of enzyme to the active sites of the biopolymer chains, thereby possibly enhancing the resistance against hyaluronidase digestion.

### 3.4. Cell Viability

**Figure 4:**
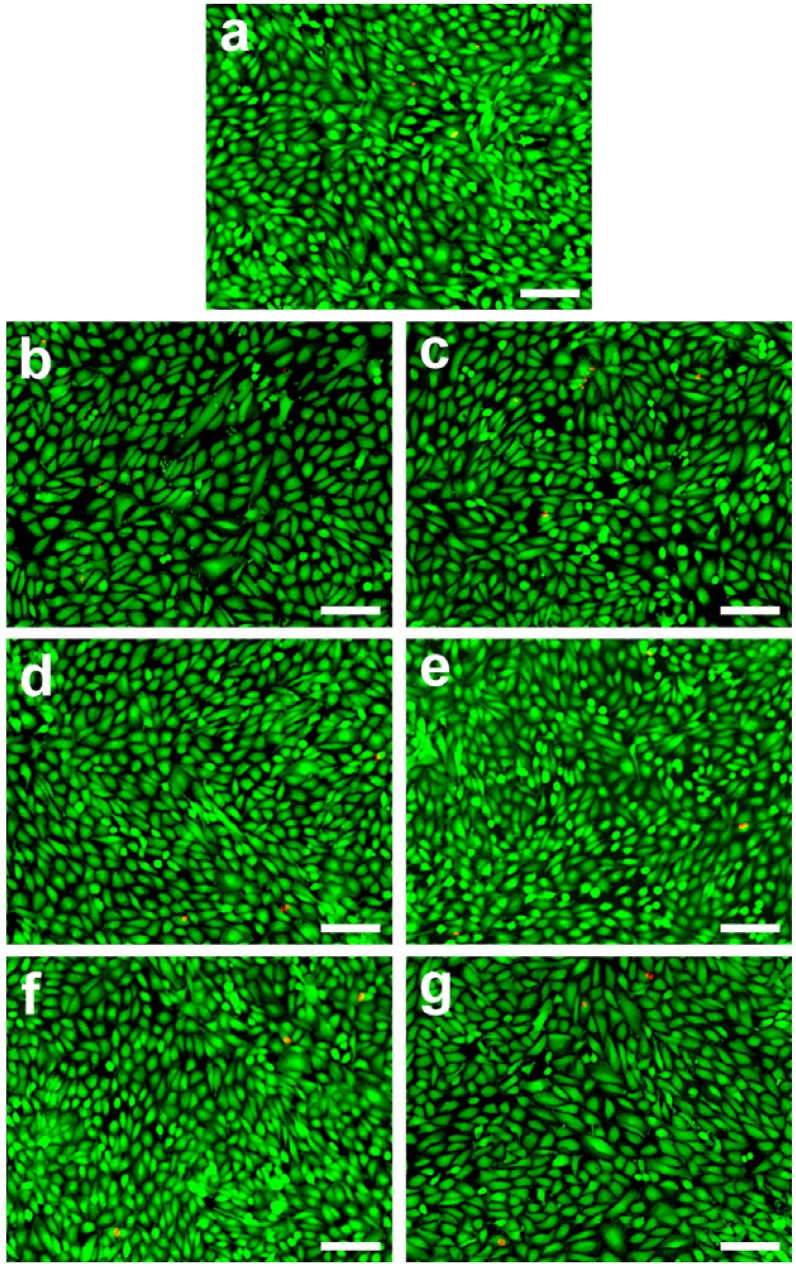
Cell viability of ARPE-19 cultures was determined by staining with Live/Dead Viability/Cytotoxicity Kit in which the live cells fluoresce green and dead cells fluoresce red. Fluorescence images of cells in (**a**) controls (without materials) after exposure to various EDC cross-linked HA membranes (**b**) A70W30; (**c**) A75W25; (**d**) A80W20; (**e**) A85W15; (**f**) A90W10; and (**g**) A95W05 for 3 days at 37 °C. Scale bars: 50 μm.

**Figure 5:**
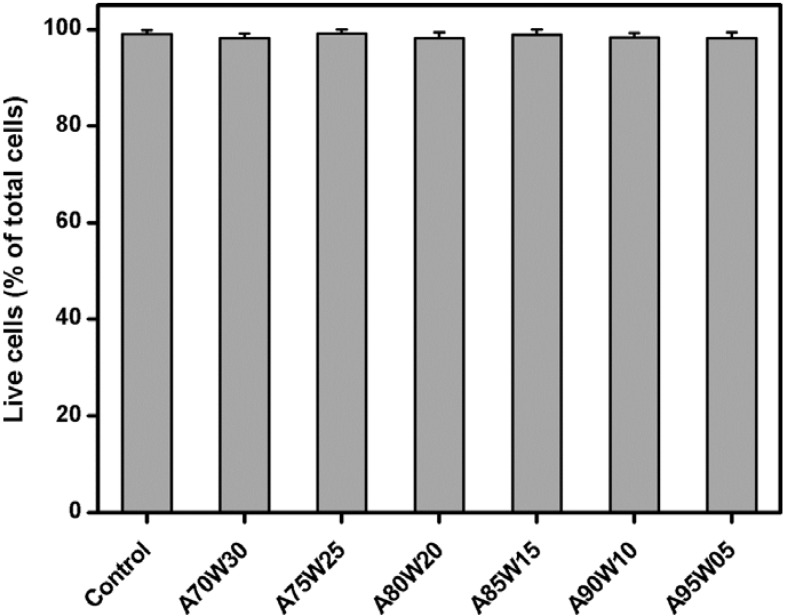
Mean percentage of live cells in the ARPE-19 cultures exposed to various EDC cross-linked HA membranes as measured by the Live/Dead assay. Values are mean ± SD (*n* = 3). No significant difference in the cell viability was observed between the control (without materials) and all the experimental groups (*P* > 0.05).

Figure 4 shows representative photographs of ARPE-19 cells labeled with Live/Dead stain, where the live cells fluoresce green and the dead cells fluoresce red. The cultures from the control groups maintained good viability and appeared healthy. Prominent green fluorescence was also detected in the A70W30, A75W25, A80W20, A85W15, A90W10 and A95W05 groups. Only a few red-stained nuclei were noted for RPE cells exposed to various EDC cross-linked HA membranes, indicating negligible cytotoxicity. The quantitative analysis of mean percentage of live cells was performed following the Live/Dead assay, and the results are shown in Figure 5. Similar levels of cell viability (98.1%–99.1%) were observed between the control and all the experimental groups (*P* > 0.05). Our data demonstrates that the ARPE-19 cells are able to maintain typical epithelial-like morphology and relatively high viability after a 3-day exposure to the test samples prepared by the cross-linking in the presence of solvent mixtures containing 70–95 vol % of acetone, suggesting good cytocompatibility of these EDC treated HA hydrogels.

Live/Dead assays are often utilized to examine the *in vitro* biocompatibility of chemically modified HA materials. Nakaji-Hirabayashi *et al.* demonstrated that the HA cross-linked by the peptide carrying oligohistidine segments at the both termini could be used in neural tissue engineering without cell toxicity [41]. Jin *et al.* reported that bovine chondrocytes incorporated in the HA grafted with a dextran-tyramine conjugate hydrogels were viable and able to enhance cell proliferation and matrix production [42]. Recently, Bae *et al.* showed that the photo-cured hydrogels composed of 2-aminoethyl methacrylate and HA had good biocompatibility for use as scaffolds for bone tissue regeneration [43]. As an ophthalmic biomaterial, HA has been extensively injected into the anterior chamber for protecting the corneal endothelium. In 2008, Spitzer *et al.* have evaluated the biocompatibility of a concentrated hydrophilic steroid formulation from commercially available HA gels as a potential adjunct in glaucoma surgery and concluded that the drug delivery carriers are not detrimental to both the human tenon fibroblasts and RPE cells [44]. However, to the best of our knowledge, the effect of carbodiimide treated HA materials on the compatibility with RPE cultures is yet to be determined. In this study, irrespective of the solvent composition (in the range of 70%–95%), the cross-linked HA hydrogel membranes are non-toxic towards human RPE cell lines.

### 3.5. Pro-Inflammatory Cytokine Expression

**Figure 6:**
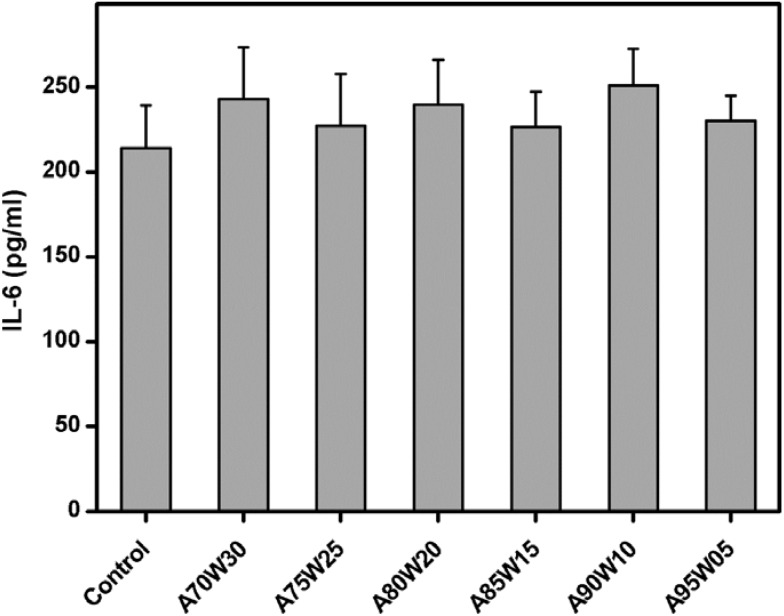
Level of IL-6 released from ARPE-19 cultures after exposure to various EDC cross-linked HA membranes for three days. The cultures in absence of HA materials served as control groups. Values are mean ± SD (*n* = 4). No significant difference in the IL-6 level was observed between the control and all the experimental groups (*P* > 0.05).

After a 3-day exposure of ARPE-19 cells to various EDC cross-linked HA membranes, the pro-inflammatory cytokine production is shown in Figure 6. The expression level of IL-6 in the control, A70W30, A75W25, A80W20, A85W15, A90W10, and A95W05 groups was 214.1 ± 25.2, 243.0 ± 30.3, 227.1 ± 30.7, 239.7 ± 26.3, 226.4 ± 20.8, 251.0 ± 21.2, and 230.2 ± 14.8 pg/mL, respectively. These values did not show a statistically significant difference (*P* > 0.05), suggesting that the HA materials prepared by the cross-linking in the presence of acetone/water mixtures with varying solvent compositions do not stimulate pro-inflammatory cytokine IL-6 secretion in human RPE cell line cultures. Given that EDC cross-linking involves the formation of essentially interchain ester bonds, with no added bridging moieties [45], we have employed this technique to develop biocompatible delivery carriers made of various biomaterials such as HA [22], gelatin [21], amniotic membrane [27], gelatin/poly(*N*-isopropylacrylamide) [32], and gelatin/chondroitin sulfate [24]. The data from Tomihata *et al.* showed that the carbodiimide cross-linked HA films implanted subcutaneously in the backs of Wistar rats elicited no significant inflammatory reaction to the surrounding tissue [46]. In accordance with their findings, our study indicated that the EDC treated HA implants have good ocular tolerability in the anterior chamber of a rabbit eye model without causing adverse inflammatory reaction [18]. Here, we further investigated the relationship between inflammation and solvent composition-mediated cross-linking by focusing on the RPE cell secretion of IL-6 in response to chemically modified HA hydrogel membranes. In the presence of solvent mixtures containing 70–95 vol % of acetone, the treatment of HA materials with EDC does not affect pro-inflammatory cytokine expression.

### 3.6. Cell Proliferation

**Figure 7:**
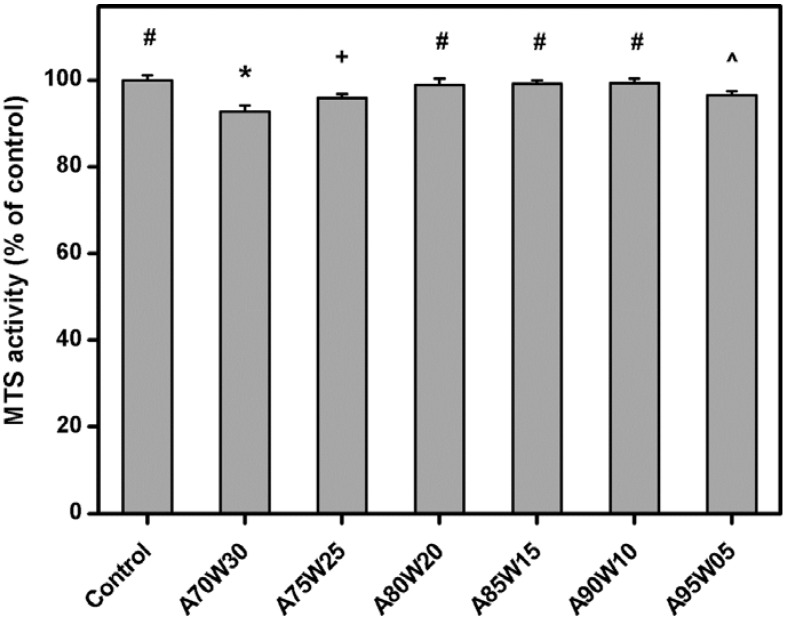
Cell proliferation assay of ARPE-19 cultures incubated for three days at 37 °C with various EDC cross-linked HA membranes. Results are expressed as percentage of controls (MTS activity of cells cultured in the absence of materials). Values are mean ± SD (*n* = 5). *P < 0.05 *vs.* all groups; ^#^*P* < 0.05 *vs.* A70W30, A75W25, and A95W05 groups; ^+^*P* < 0.05 *vs.* all groups, except A95W05; ^^^*P* < 0.05 *vs.* all groups, except A75W25.

Figure 7 shows the results of human RPE cell proliferation after incubation with various EDC cross-linked HA membranes for three days. Similar levels of mitochondrial dehydrogenase activity (MTS activity) were observed in the control, A80W20, A85W15, and A90W10 groups and not statistically different (*P* > 0.05). These findings suggest that the HA membranes prepared by the cross-linking in the presence of acetone/water mixtures of different acetone concentrations (80–90 vol %) do not affect the metabolic activity of the ARPE-19 cultures. However, the cells exposed to the test samples from both the A75W25 (95.8 ± 1.0%) and A95W05 (96.5 ± 0.9%) groups were less metabolically active and had a lower density than those from all the other groups (*P* < 0.05). In particular, in the A70W30 groups, the MTS activity was significantly reduced by about 7.3% (*P* < 0.05) as compared to that of the control groups. Our data demonstrates that the solvent composition-mediated cross-linking of biomaterials may play an important role in the regulation of RPE cell growth.

In the field of corneal regenerative medicine, gelatin materials have been used to fabricate biopolymer-based carriers for delivery of tissue-engineered cell sheets [47,48,49]. The charge and the degree of polymerization of gelatin are found to affect its affinity for human corneal endothelial cells [28]. In this study, the HA samples with a low cross-linking degree (*i.e.*, water content ≥ 83.4%) have a slight inhibitory effect on ARPE-19 proliferation, which may reflect the alteration in the ionic interaction between the medium nutrients and polysaccharide biomaterials. EDC cross-linking is a process of linking between two sites through the consumption of free carboxylic acid groups in HA molecules. Therefore, the HA hydrogel membranes with low extent of cross-linking theoretically contain larger amounts of negatively charged functional groups to enhance ionic interactions and restrict nutrient availability for cell growth. This may explain the findings of the current study.

## 4. Conclusions

Here, we have optimized the solvent composition-mediated cross-linking of HA materials by the determination of structural stability, enzymatic degradability and cytocompatibility. After the EDC treatment in the presence of an acetone/water mixture (85:15, v/v), the HA hydrogel membranes have the lowest equilibrium water content, the highest stress at break and the greatest resistance to hyaluronidase digestion. Irrespective of the solvent composition (in the range of 70%–95%), the cross-linked HA hydrogel membranes are compatible with human RPE cell lines without causing toxicity and inflammation. However, it should be noted that the test samples prepared by cross-linking, in the presence of acetone/water mixtures containing 70, 75, and 95 vol % of acetone, slightly inhibit the metabolic activity of viable ARPE-19 cultures. In summary, the water content, mechanical strength and RPE cell proliferative capacity strongly depends on the solvent composition for carbodiimide cross-linking of HA materials.
